# Kupffer Cells: Important Participant of Hepatic Alveolar Echinococcosis

**DOI:** 10.3389/fcimb.2020.00008

**Published:** 2020-01-29

**Authors:** Yumei Liu, Fengming Tian, Jiaoyu Shan, Jian Gao, Bin Li, Jie Lv, Xuan Zhou, Xuanlin Cai, Hao Wen, Xiumin Ma

**Affiliations:** ^1^State Key Laboratory of Pathogenesis, Prevention and Treatment of High Incidence Diseases in Central Asia, Clinical Medical Research Institute, First Affiliated Hospital of Xinjiang Medical University, Urumqi, China; ^2^College of Basic Medicine of Xinjiang Medical University, Urumqi, China

**Keywords:** hepatic alveolar echinococcosis, liver fibrosis, KCs, HSCs, cytokine

## Abstract

**Aims:** Kupffer cells (KCs) are the liver-resident macrophages and play a leading role in the regulation of liver homeostasis in physiological conditions and in pathology. The study aims to investigate the anti-echinococcosis effect of KCs and the effects of hepatic stellate cells (HSCs) activation in the progression of liver fibrosis in hepatic alveolar echinococcosis (hepatic AE).

**Methods:** Hematoxylin—eosin (H&E) and Masson staining were used to assess the pathological inflammatory changes and collagen deposition, respectively. Immunohistochemistry and qRT-PCR were used to detect the number of aggregates of KCs, the expression of cytokines and activation of HSCs.

**Results:** In the close group, H&E staining showed that the normal lobular structure was destroyed and inflammatory infiltration around the lesion could be observed, and Masson staining showed that blue collagen fibers were clearly deposited near the portal area. IHC showed that KCs surface markers CD68 and CD163, cytokine iNOS and Arg-1 were positively expressed in the vicinity of inflammatory lesions. qRT-PCR indicated that TNF-α, IL-10, and TGF-β1 secreted by KCs were significantly higher than those in the distance group (*P* < 0.01). It is worth noticing that the expression levels of anti-inflammatory cytokines were slightly higher than that of pro-inflammatory cytokines. Both IHC and qRT-PCR results showed that HSCs activation markers, the expression of α-SMA and Desmin significantly increased.

**Conclusions:** Our research indicates that KCs have immune-protective effect of anti-echinococcosis and promote liver fiber repair, and it also suggests that they have potential therapeutic value for patients with hepatic AE.

## Introduction

Alveolar echinococcosis (AE), caused by *Echinococcus multilocularis*. is characterized by a large multilocular cyst with a jelly-like substance, instead of clear hydatid fluid. As most cases involve the liver, patients may suffer from hepatomegaly and recurrent jaundice (Menghi et al., 2017). Cysts localize first in the liver, and in the early stages, the infection is generally asymptomatic (Arrechea Irigoyen et al., 2008). As the growth pattern of the cyst is similar to a malignant tumor, the WHO has proposed that a clinical classification that is similar to TNM (Tumor, Node, Metastases) classification of tumors. Such classification is a necessary tool when making therapeutic decisions for the treatment of this disease (Kern et al., 2006). AE is a serious life-threatening chronic helminthiasis caused by *E. multilocularis*. It mostly occurs in the liver and is known to be slowly progressive but often, a fatal disease. It is estimated that nearly 2 billion people worldwide are infected with worms (Hotez et al., 2008) and about 200 million cases are *echinococcosis*, of which, 0.3% are caused by AE (Craig et al., 2017). Some experimental studies, including experimental studies on infected mice and immunological studies on AE patients, have revealed that complex host-parasite interaction occurs in the process of *E. multilocularis* infection (Wang and Gottstein, 2016). The variability and severity of the clinical manifestations of this parasitic disease are related to the duration and degree of infection (Mezioug and Touil-Boukoffa, 2012).

Liver fibrosis is one of the main pathological changes in the progression of hepatic AE. When acute liver injury occurs, the accumulation of extracellular matrix (ECM) secreted by fibroblasts is a normal feature of wound healing during acute inflammation. However, under most chronic or persistent inflammatory injuries, such as alcoholic hepatitis, viral hepatitis, autoimmune liver disease, and parasitic diseases, this mechanism of liver tissue repair is abnormally regulated and leads to irreversible fibrosis, even eventually develops into cirrhosis and liver cancer.

The inflammatory stimulation of *E. multilocularis*, an invader of the liver environment, promotes the activation and proliferation of a large number of Kupffer cells (KCs). KCs are specialized macrophages that reside in the liver and belong to the mononuclear phagocyte system. In addition to the phagocytic capacity, they can immediately respond to non-specific defense responses and have the ability to recruit other immune cells. These cells are highly malleable and can be altered according to changes in the microenvironment of the liver, both in morphology and function (Tacke and Zimmermann, 2014; Wynn and Vannella, 2016). In acute hepatic inflammatory injury, KCs release the pro-inflammatory cytokine inducible nitric oxide synthase (iNOS) through direct contact between cells and hepatocytes, and then release NO to effectively kill pathogens (Elchaninov et al., 2019). In order to limit the continuous stimulation of *E. multilocularis* and protect the stability of the liver environment, KCs secrete a large amount of profibrogenic cytokine transforming growth factor-β1 (TGF-β1), to promote the activation and proliferation of hepatic stellate cells (HSCs), a marker of liver fibrosis activation and leading to the occurrence and development of liver fibrosis (Lee and Friedman, 2011; Tosello-Trampont et al., 2011; Beljaars et al., 2014; Sica et al., 2014). In turn, HSCs further promote the proliferation and differentiation of KCs through paracrine effects. When patients with hepatic AE show clinical symptoms, most of them are in the middle or late stages of the disease, often accompanied with liver fibrosis and it is irreversible. Therefore, in the middle or late stages of tissue repair, KCs highly express anti-inflammatory surface marker CD163, upregulate the secretion of cytokine Interleukin-10 (IL-10), arginasing synthesis of polyamines (Arg-1), and promoting angiogenesis etc. to show its anti-inflammatory influence and repair effects (Fabriek et al., 2009). The main purpose of this study is to investigate the anti-*alveolar echinococcosis* effect of KCs in hepatic AE accompanied with liver fibrosis, and aims to evaluate KCs' potential therapeutic value in the treatment of liver fibrosis caused by persistent AE infection.

## Materials and Methods

### Patients

A total of 33 diagnosed hepatic AE patients were enrolled in the First Affiliated Hospital of Xinjiang Medical University from March 2017 to March 2019, including 17 males and 16 females with an average age of 40.61 years old (9–65 years old). Inclusion criteria was: The diagnosis of AE was in accordance with the classification criteria established by the World Health Organization (WHO) unofficial working group (Kern et al., 2006), confirmed by surgery and post-operative pathology. Patients with infectious diseases (bacteria, viruses, etc.), malignant tumors, rheumatic immune diseases, *cystic echinococcosis* or other parasitic diseases, and who took non-caries Body anti-inflammatory drugs, hormone drugs, psychotropic drugs, etc. were excluded (Kern et al., 2006). At the same time, 33 healthy age-matched controls from blood bank donors in the hospital were selected, including 17 males and 16 females, with an average age of 41.50 years (19–56 years old). Blood tests, electrocardiogram and B-ultrasound all showed no obvious abnormalities. Prior written and informed consent were obtained from patients, the minors (<18 years old) who participated in the study, had informed consent signed by their parents/legal guardians. This study conformed to the approved institutional guidelines and was approved by the Ethical Committee of Xinjiang Medical University.

### Tissue Collection and Biochemical Analysis

In hepatic AE patients, the liver tissues were taken within 2 cm of the lesion by surgery for the close group, whereas the liver tissues were taken 2 cm outside the lesion for the distance group. Part of the liver tissues were fixed with 10% formaldehyde, paraffin-embedded and sectioned for 3 um successively for H&E staining, Masson staining and immunohistochemistry. The other part of liver tissue was frozen in the refrigerator at −80°C for qRT-PCR detection. Blood, both in hepatic patients and healthy age-matched controls, was obtained for the measurement of biochemical parameters using standard methods. Serum levels of alanine aminotransferase (ALT), aspartate aminotransferase (AST), alkaline phosphatase (ALP), glutamyl transpeptidase (GGT), total bilirubin (TBIL), indirect bilirubin (IBIL), and direct bilirubin (DBIL) were determined by an automatic blood biochemical analyzer (Beckman Counter LX20, USA).

### H&E, Masson Staining, and Immunohistochemistry

Tissue slices were prepared at a 3 μm thickness and stained with hematoxylin-eosin(H&E), Masson's trichrome, or immunohistochemically (IHC) according to standard procedures. After H&E staining, observations were made and pictures were taken under the microscope (OLYMPUS BX43, Japan). In reference to the chronic hepatitis GS score, two experienced pathologists blindly developed a pathology score to assess the severity of liver inflammatory lesions in patients with hepatic AE by a microscope (OLYMPUS BX43). After Masson staining, observations were made and pictures were taken under the microscope (OLYMPUS BX43) Afterwards, the sections were assessed for the METAVIR fibrosis score, as adapted from the study by Zhang et al. (2016). Detailed as follows,

- 0 score: Fibrosis free;
- 1 score: fibrosis is limited to the portal area;
- 2 score: fibrosis is not limited to the portal area, a small part of the development to the hepatic lobules;
- 3 score: fibrosis into the central vein of the hepatic lobules;
- 4 score: false Lobular formation, lesions even develop into cirrhosis.

For immunohistochemistry, the sections were incubated with primary antibodies at 4°C overnight. The sections were then incubated with the secondary antibody (biotinylated goat anti-rabbit IgG) (Mavision™, Maxim, China) for 30 min. The signal was amplified using either streptavidin—biotin complexes conjugated with peroxidase and 3, 3′ diaminobenzidine, or aminoethyl carbazole (Mavision™, Maxim, China). Next, the sections were counterstained with hematoxylin and mounted using cover slips. Observations were made and pictures were taken under the microscope (OLYMPUS BX43, Japan).

### RNA Extraction and Real-Time PCR

Total RNA was isolated from homogenized liver tissues using a TRIzol™ isolation kit (Takara Bio, Dalian, China) following the manufacturer's protocol. The cDNA was synthesized by using Primer Script RT kit (Takara Bio, Dalian, China). Prime Script™ RT reagent kits, along with SYBR Green Realtime PCR Master Mix and Permix Ex Taq (Takara Bio), according to the manufacturer's instructions. The primers for GAPDH, TNF-α, IL-10, TGF-β1, α-SMA, and Desmin were synthesized by Sangon Biotech (Shanghai, China). Real-Time PCR was operated on ABI Prism 7500 Sequence Detection System (BioRad, Life Science Research, Hercules, CA, USA). PCR conditions were as follows: one cycle at 95°C for 30 s, 40 cycles at 95°C for 5 s, at 64°C for 30 min. All reactions were performed in triplicate for each sample. The 2^−ΔΔCT^ method was used to calculate relative concentration of each target by standardizing to internal GAPDH level.

### Statistical Analysis

Data was shown as the means ± standard error of mean (SEM). They were analyzed by SPSS 21.0 (IBM, Chicago, IL, USA) or GraphPad Prism 7.0 software (GraphPad Software, San Diego, CA, USA). Student's *t*-test was performed to determine differences between two groups. *P* < 0.05 indicated statistical significance.

## Results

### Biochemical Parameters in Patients With Hepatic AE

Notably, serum levels of ALT, AST, GGT, ALP, IBIL, and DBIL were significantly higher in patients with hepatic AE than those in healthy controls (shown in Table 1). It indicated that the normal function of hepatocytes was impaired, and the liver microenvironment was severely imbalanced in patients with hepatic AE.

**Table 1:** Biochemical parameters in Hepatic AE group and Healthy control group (Mean ± SD).

**Index**	**HAE (*n* = 33)**	**Control (*n* = 33)**
Age (average)	40.61	41.50
Sex (male:female)	1.06:1	1.06:1
ALT (U/L)	65.21 ± 44.12^****^	23.96 ± 10.48
AST (U/L)	47.36 ± 27.52^****^	23.87 ± 5.72
ALP (U/L)	139.77 ± 60.74^****^	65.38 ± 13.27
GGT (U/L)	82.66 ± 56.76^****^	26.59 ± 12.69
TBIL (umol/L)	15.14 ± 8.26	14.83 ± 4.37
I-BIL (umol/L)	9.02 ± 4.99^**^	12.13 ± 3.71
DBIL (umol/L)	6.70 ± 5.15^***^	2.70 ± 0.77

**** *P < 0.0001*,

*** *P < 0.001*,

** *P < 0.01*.

### Pathological Changes Were Observed in Liver Lesions of Patients With Hepatic AE

**Figure 1:**
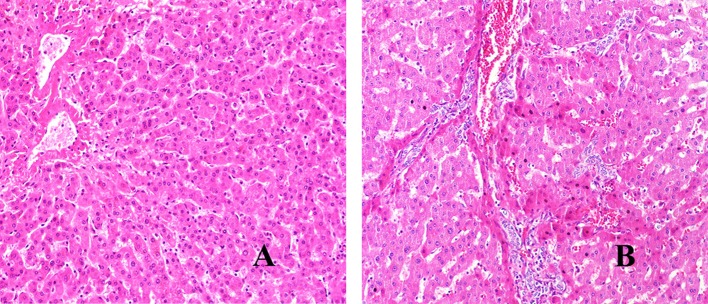
H&E staining of liver lesions in patients with hepatic AE (magnification, ×200). **(A)** Distance group. **(B)** Close group.

**Figure 2:**
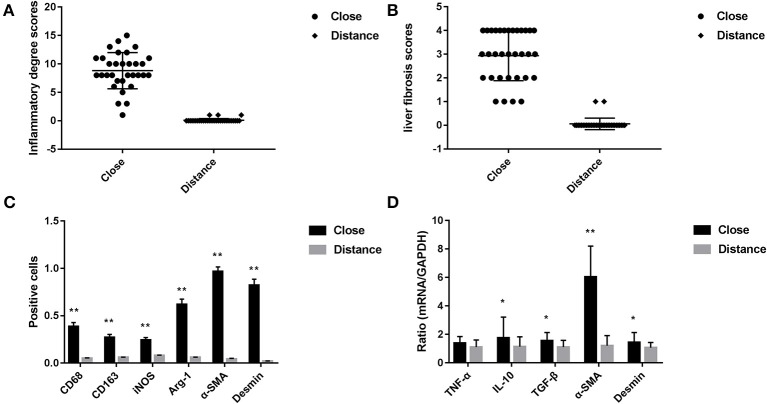
**(A)** Pathological score of liver tissue inflammatory changes in patients with hepatic AE (after H&E staining, score criteria refers to the Table 2). **(B)** Liver fibrosis score in patients with hepatic AE (after Masson staining, refers to METAVIR scoring standard). **(C)** Immunohistochemical positive cells area of liver tissue in patients with hepatic AE (magnification, ×400). KCs surface markers CD68 and CD163, pro-inflammatory cytokine iNOS, anti-inflammatory cytokine Arg-1 were differentially expressed in immunohistochemical staining between the two groups. The difference in the HSCs activation markers α-SMA and Desmin between the two groups (**P < 0.01, compared with Distance group). **(D)** Gene expression levels of KCs and HSCs activation (*P < 0.05, **P < 0.01, compared with Distance group).

We have used H&E staining to observe the pathological morphology of livers. The close group showed that the normal lobular structure of liver tissue was destroyed. Cell edema, cytoplasm loose, a large amount of congestion in the central vein of the liver, and the hepatic sinus was clearly dilated, some inflammatory lesions were visible, and a large number of inflammatory cells infiltrated around them (shown in Figure 1). In contrast, the distance group showed that the hepatic lobule structure of the liver tissue was normal, and the central vein was visible inside. The hepatocytes were arranged radially around the central vein, the structure of the hepatic sinus was clear and there was no pathological change. Combined with the chronic hepatitis GS score, two pathologists blindly assessed the severity of liver inflammatory lesions in patients with hepatic AE (severity criteria details shown in Table 2, results shown in Figure 2A).

**Table 2:** Pathological scores of liver lesions' inflammatory changes in patients with hepatic AE.

**Cholestasis**	**Calcification**	**Vesicles (number)**	**Necrotic liquefaction cavity (number)**	**Inflammatory cells infiltration**	**Scores**
	Calcification zone could be observed	≥16	≥10	Confluent lesion infiltration	4
	Confluent calcification	11–15	7–9	More than 4 lesions infiltration	3
+	Irregular calcification	6–10	4–6	2–4 lesions infiltration	2
	Point calcified particles	1–5	1–3	Single lesion infiltration	1
_	No obvious change	0	0	No obvious change	0

*After H&E staining, pathological score criteria was drawn up for evaluating the inflammatory changes in liver lesions in patients with hepatic AE (results were shown in Figure 2A). Among them, the vesicle count was observed in at least 3 different fields and the necrotic liquefaction cavity was the sum of the entire pathological section of view under low-power microscope (magnification, ×100)*.

**Figure 3:**
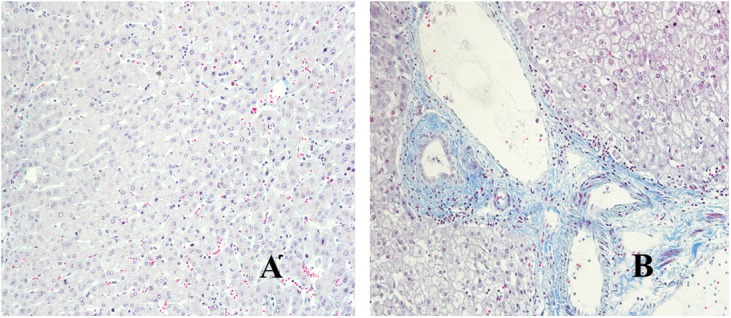
Masson staining of liver lesions in patients with hepatic AE (magnification, ×200). **(A)** Distance group. **(B)** Close group.

We have used Masson staining to observe the collagen deposition. Masson staining indicated that there was no inflammatory cell infiltration in the liver tissue portal area, and little or no collagen fiber tissue in Distance group. However, in the close group, the normal structure of the hepatic lobules disappeared, the hepatocytes were disorderly arranged, the volume became larger, nuclear dissolution occurred, and the blue collagen fibrous tissue was clearly proliferated and deposited (shown in Figure 3). Referring to METAVIR scoring criteria, the fibrosis score of patients with hepatic AE as shown in Figure 2B.

### KCs Secrete Some Cytokines, and Anti-inflammatory Cytokines Were Expressed at Higher Levels Than Pro-inflammatory Cytokines in Patients With Hepatic AE

**Figure 4:**
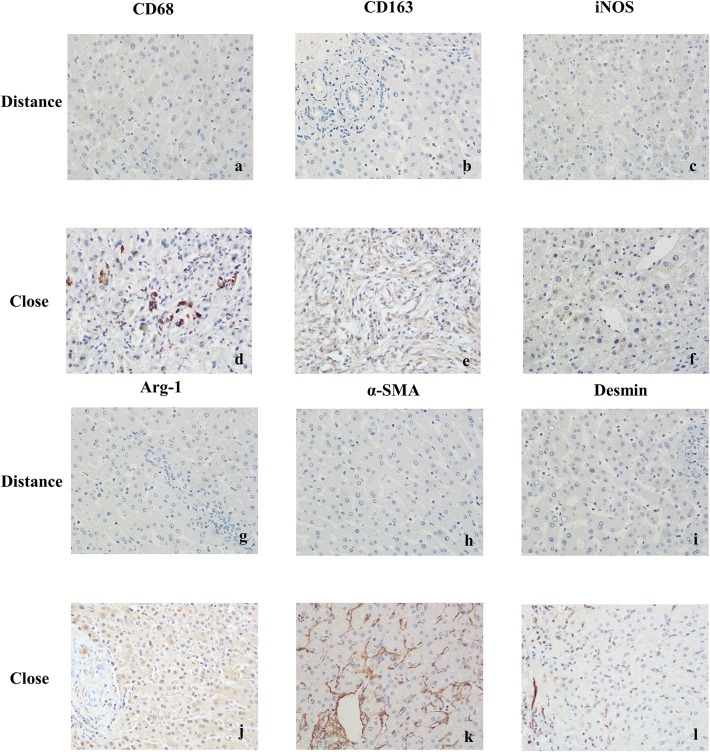
Immunohistochemical staining results of liver tissues in patients with hepatic AE (magnification, ×400). **(a)** CD68 was weakly positive or negative in KCs cytoplasm (Distance group). **(b)** CD163 was weakly positive or negative in KCs cytoplasm (Distance group). **(c)** iNOS was weakly positive or negative in KCs cytoplasm (Distance group). **(d)** Strong positive expression of CD68 in KCs cytoplasm (Close group). **(e)** Strong positive expression of CD163 in KCs cytoplasm (Close group). **(f)** When exposed to inflammatory stimuli, KCs secreted the pro-inflammatory cytokine iNOS, strong positive expression of iNOS in KCs cytoplasm (Close group). **(g)** Arg-1 was weakly positive or negative in KCs cytoplasm (Distance group). **(h)** α-SMA was weakly positive or negative in cytoplasm of resting HSCs (Distance group). **(i)** Desmin was weakly positive or negative in cytoplasm of resting HSCs (Distance group). **(j)** In the continuous parasite stimulation, KCs secreted the anti-inflammatory cytokineArg-1, strong positive expression of Arg-1 in KCs cytoplasm (Close group). **(k)** Strong positive expression of α-SMA in cytoplasm of activated HSCs (Close group). **(l)** Strong positive expression of Desmin in cytoplasm of activated HSCs (Close group).

CD68 is commonly used as a surface marker for KCs in human (Sica et al., 2014; Koyama and Brenner, 2017), during liver tissue self-repair stage, KCs highly express CD163 (You et al., 2013; Bala et al., 2016; Björklund et al., 2018). Patients with hepatic AE are generally in the middle, even late stages of the disease. At this point, liver fibrosis has formed and the liver is in the period of tissue repair. Therefore, CD68 and CD163 were used as surface markers for KCs in our study. In IHC (Antibodies in IHC shown in Table 3), CD68 showed a brown mass around the liver lesions, and the positive results were mainly expressed in the cytoplasm of KCs. Because the vesicles of the *echinococcosis* continued to expand and compress the liver tissue, the inflammation continuously stimulated the liver tissue, KCs clustered around the lesions and actively exerted anti-inflammatory effects. While the distance group showed lower expression (*p* < 0.01; Primer sequence shown in Table 4, results shown in Figures 2C, 4a,d). Meanwhile, the positive result of CD163 was expressed as brownish yellow particles in the cytoplasm of KCs (Figures 4b,e), which is consistent with the results of CD68. When liver is damaged or infected, iNOS—a kind of catalytic enzyme, is released by KCs to promote the inflammatory (Anavi et al., 2015; Cinar et al., 2016). After activation, a large amount of NO is produced, which enhances the degree of oxidative stress in the body and promotes the expression of pro-inflammatory substances by inflammatory cytokines such as TNF-α etc., which simultaneously expresses and secretes pro-inflammatory substances to clear pathogens. In protein level, iNOS was positive in the cytoplasm or nucleus of KCs around the lesion while the distance group showed less positive cells (*p* < 0.01; Figures 2C, 4c,f). In gene level, TNF-α was also at a high expression level than that in the distance group (*p* < 0.05; Figure 2D). In contrast to the pro-inflammatory effect of iNOS and TNF-α, KCs also can secrete an amount of cytokine Arg-1 and cytokine IL-10 (Altamirano-Barrera et al., 2017; Kim et al., 2017; Campana et al., 2018), they can down-regulate iNOS and TNF-α activity reducing cell apoptosis (Lisi et al., 2017). In protein level, Arg-1 was mostly distributed in the cytoplasm of macrophages around the lesion tissue and appeared as brownish yellow particles (Figures 4g,j). In gene level, IL-10 was also at a high expression level than that in the distance group (*p* < 0.05; Figure 2D). Interestingly, we found that the anti-inflammatory cytokines were higher than pro-inflammatory cytokines both in the protein expression and gene levels. A possible reason for this could be due to the liver tissue already being at the anti-inflammatory and self-repairing stage, when the patients with hepatic AE received surgery. At the same time, liver fibrosis had already formed and gradually progressed.

**Table 3:** Antibodies used for immunohistochemistry.

**Antibody**	**Dilution factor**	**Company**
Anti-CD68 Antibody	1:200	Bioss, Beijing, China
Anti-CD163 Antibody	1:200	Bioss, Beijing, China
Anti-iNOS antibody	1:1000	Abcam, Cambridge, UK
Rabbit Anti-Arg-1 antibody	1:200	Bioss, Beijing, China
Rabbit Anti-alpha-SMA antibody	1:400	Affinity, Cincinnati, US
Rabbit Anti-Desmin antibody	1:200	Affinity, Cincinnati, US

**Table 4:** Primer sequence.

**Gene (human)**	**Primer sequence**
TNF-α	F: TGCTCCTCACCCACACCAT
	R: GGAGGTTGACCTTGGTCTGGTA
IL-10	F: GGGAGAACCTGAAGACCCTCA
	R: TGCTCTTGTTTTCACAGGGAAG
TGF-β1	F: CAATTCCTGGCGATACCTCAG
	R: GCACAACTCCGGTGACATCAA
α-SMA	F: TTGAGAAGAGTTACGAGTTG
	R: GGACATTGTTAGCATAGAGG
Desmin	F: AGCCAGGCCTACTCGTCCAGCCA
	R: CCGCCCGACGTGCGCGACACCTG
GAPDH	F: CATCCACTGGTGCTGCCAAGGCTGT
	R: ACA ACCTGGTCCTCAGTGTAGCCCA

*F, Forward; R, Reverse*.

### KCs Secrete a Large Amount of Cytokine TGF-β1 Trigger HSCs Activation and Proliferation in Liver Lesions of Hepatic AE Patients

In order to protect the stability of the liver microenvironment and repair the liver damage during the continuous stimulation of *E. multilocularis*, KCs secrete a large amount of pro-fibrogenic cytokine TGF- β1, which promotes the activation and proliferation of HSCs. The activation of HSCs is central for liver fibrogenesis, because these cells transdifferentiate into myofibroblasts and represent the major extracellular matrix producing cells in the liver (Tsuchida and Friedman, 2017). TGF- β1 positively regulates the activation of HSCs. In order to detect the changes of TGF-β1 at mRNA level in the liver lesions of hepatic AE patients, we performed qRT-PCR assay. As shown in Figure 2D, the mRNA level of TGF-β1 in the liver lesions was significantly elevated in the close group than that in the distance group (*P* < 0.05). α-SMA, Desmin are the surface markers of HSCs activation (Ding et al., 2014; Zhang et al., 2018; Inzaugarat et al., 2019), mainly express in the cytoplasm of the vascular wall or in the cytoplasm of activated HSCs. We performed IHC and qRT-PCR assay to assess the protein and gene levels, respectively. In the close group, IHC indicated there was strong expression of α-SMA and Desmin reaction in lesion tissues, increased brown-yellow staining could be observed in the portal area and fiber compartment (shown in Figures 4k,l), it suggested that HSCs were in a proliferating stage. Compared with the distance group, the expression of α-SMA and Desmin was significantly increased (shown in Figures 2C, 4h,i; *P* < 0.01). As shown in Figure 2D, the mRNA levels of α-SMA in the liver was significantly elevated in the close group than that in the distance group (*P* < 0.01). Meanwhile the mRNA levels of Desmin in the liver lesions was significantly elevated in the close group than that in the distance group (*P* < 0.05), the difference was statistically significant.

## Discussion

Liver is the main parasitic organ infected with hydatid cyst. Its pathological structure is a collection of numerous small vesicles with a diameter of 0.1–1.0 cm. Its general view is a single large block, which is a pale yellow or white vesicle-like mass, with a hard texture and unclear boundaries with surrounding tissues. Liver fibrosis is a protective pathological process triggered by continuous stimulation of *echinococcosis*. Early liver fibrosis can be reversed and collagen can limit the expansion of the worm as well as repair damaged liver tissue. The situation of fibrosis, in the middle and late stages, starts to deteriorate, reaching an irreversible level, thus can further develop into cirrhosis and liver cancer. It is currently difficult to completely remove echinococcal vesicles by surgery. Therefore, timely administration of anti-fibrosis therapy is an important measure to hinder the progress of the disease. Our study firstly performed pathological scoring on liver lesions of patients with hepatic AE, including common items such as inflammatory cell infiltration, cholestasis etc. and the number of vesicles in the disease was also included to assess the severity of liver pathological changes in patients with hepatic AE. At the same time, liver fibrosis severity was evaluated by METAVIR liver fibrosis score. Both scores indicated a high degree of liver inflammation, a severe degree of fibrosis, and a small number of patients have even reached the level of cirrhosis.

A large number of studies (Lin et al., 2009; Tacke and Kurts, 2011) suggested that bone marrow monocyte-derived macrophages (MoMFs) contributed significantly to parasitic liver fibrosis. However, KCs, the liver-resident macrophages distinguished from the macrophage recruited from peripheral blood, were also shown to contribute significantly to the progression of liver fibrosis, and macrophages recruited from peripheral blood. Wang et al. (2013) pointed out that the liver lesions of patients with echinococcosis were similar to those observed in the 180th day after infection in experimental mice. Typical chronic granuloma and fibrosis changes existed around the vesicles. In an area of the liver, far from the lesion, a large number of lymphocytes infiltrated the portal vein causing the degeneration and necrosis of some of the hepatocytes; which resulted in the proliferation and differentiation of a large number of KCs. In this study, H&E staining and Masson staining were used to evaluate the pathological changes and fibrosis of liver lesions in patients with hepatic AE. The pathologic manifestations, described in previous studies, in liver lesion tissues of patients with hepatic AE were verified. CD68 and CD163 were selected as the surface markers of KCs in immunohistochemical staining to observe the number of activated KCs proliferation (Bala et al., 2016; Björklund et al., 2018). In the distance group, a small number of cells showed yellowish clumps in cytoplasm that were weakly positive for CD68, suggesting that there was still a small amount of active KCs expression in distant liver tissue. In the close group, CD68 and CD163 were positively expressed in liver lesions. It indicated that a large number of KCs were activated to accumulate around the lesions and responded to the immune response during the process of continuous stimulation of echinococcosis in the liver.

The patients, who had no clinical manifestations in the early stage of infection, most of them had reached the middle or late stage with clear fibrosis changes in the liver with hepatic AE. In our study, the liver lesions in the patients with hepatic AE showed a clear collagen deposition, presenting the middle and late stage of liver fibrosis, that is, the liver tissues were in the anti-inflammatory and self-repairing stage.

Hashimoto et al. (2013) and Sasaki et al. (2017) found that KCs, in response to liver injury, become activated and express cytokines and signaling molecules. Additionally, activated KCs display markers of M1-like macrophages or M2-like macrophages depending on the signals that they receive from their environment. Inflammation in the liver is regulated by the balance of pro-inflammatory M1 Kupffer cells and anti-inflammatory M2 Kupffer cells which was partially self-renewing in a steady state, independent of monocyte recruitment. Interestingly, in our study, the anti-inflammatory cytokines were higher than pro-inflammatory cytokines both in the protein expression and gene levels. It suggests that KCs exhibit a similar phenotype in the late stage of hepatic AE, and moreover, KCs secreted a large amount of cytokine TGF-β1 to further promote fibrosis. However, further research is needed to investigate whether it is related to peripheral blood recruited macrophages.

It is generally accepted that α-SMA and Desmin are surface markers of HSCs activation (Gibelli et al., 2008; Novo et al., 2009; Golbar et al., 2011). There is clear evidence from *in vitro* and *in vivo* studies that KCs can activate HSCs to transdifferentiate into myofibroblasts, the major collagen-producing cell type in hepatic fibrosis (Pradere et al., 2013; Tacke and Zimmermann, 2014). KCs activate HSCs via paracrine mechanisms, likely involving the potent profibrotic and mitogenic cytokines TGF-β and PDGF (Pradere et al., 2013). These profibrotic functions of KCs during chronic hepatic injury remain functionally relevant, even if the infiltration of additional inflammatory monocytes is blocked via pharmacological inhibition of the chemokine CCL2 (Baeck et al., 2012). Whether the mechanism of KCs in liver fibrosis caused by vesicular hydatid disease is consistent to other chronic liver disease, further research is needed. In our study, they were up-regulated at both protein and gene levels in patients with hepatic AE.

In summary, through investigating the activation and KCs' proliferation and HSCs' activation in patients with hepatic AE, it suggested that the long-term inflammatory reaction caused by the infection of the *echinococcosis* triggered the self-protection and self-repairing of the liver microenvironment. At the same time, it caused a large number of KCs to proliferate and update, and tended to be M2-like macrophages than M1-like macrophages, releasing a large number of anti-inflammatory cytokines to resist the stimulation of *echinococcosis*. Meanwhile KCs secreted a large amount of pro-fibrogenic cytokines to activate HSCs. KCs has a complex and highly flexible role in the anti-*echinococcosis* and self-repairing in patients with hepatic AE. It is potentially feasible to reduce the fibrosis activity of HSCs and improve its anti-fibrosis activity by targeting KCs (Han et al., 2019). However, this study mainly evaluated the liver pathological changes and degree of fibrosis to assess the severity of the patients with hepatic AE, and studied the role of KCs in liver fibrosis to evaluate its influence in hepatic AE. In the future, we will simulate the *in vivo* environment and co-culture the two cells *in vitro* to further clarify the mechanism of KCs regulating the activation of HSCs.

## Data Availability Statement

The data used to support the findings of this study are available from the corresponding author upon request.

## Ethics Statement

The studies involving human participants were reviewed and approved by Ethical Committee of Xinjiang Medical University. The patients/participants provided their written informed consent to participate in this study.

## Author Contributions

XM, HW, and FT conceptualized and design the study. XM, YL, and JG were responsible for the administrative support. JS and JL worked on the provision of the study materials or patients. BL and XC collected and assembled the data. XZ was responsible for the data analysis and interpretations. All authors wrote and approved the final manuscript.

### Conflict of Interest

The authors declare that the research was conducted in the absence of any commercial or financial relationships that could be construed as a potential conflict of interest.
